# On the Combination of Multi-Cloud and Network Coding for Cost-Efficient Storage in Industrial Applications

**DOI:** 10.3390/s19071673

**Published:** 2019-04-08

**Authors:** Goiuri Peralta, Pablo Garrido, Josu Bilbao, Ramón Agüero, Pedro M. Crespo

**Affiliations:** 1 Information and Communication Technologies Area, Ikerlan Technology Research Centre, 20500 Arrasate-Mondragón, Spain; pgarrido@ikerlan.es (P.G.); jbilbao@ikerlan.es (J.B.); 2 Department of Communications Engineering, University of Cantabria, 39005 Santander, Spain; ramon@tlmat.unican.es; 3 Electronics and Communications Department, University of Navarra (Tecnun), 20009 Donostia-San Sebastián, Spain; pcrespo@tecnun.es

**Keywords:** cost-efficiency, data availability, distributed storage, fog, Industry 4.0, multi-cloud, network coding, reliability, spot instances

## Abstract

The adoption of both Cyber–Physical Systems (CPSs) and the Internet-of-Things (IoT) has enabled the evolution towards the so-called Industry 4.0. These technologies, together with cloud computing and artificial intelligence, foster new business opportunities. Besides, several industrial applications need immediate decision making and fog computing is emerging as a promising solution to address such requirement. In order to achieve a cost-efficient system, we propose taking advantage from spot instances, a new service offered by cloud providers, which provide resources at lower prices. The main downside of these instances is that they do not ensure service continuity and they might suffer from interruptions. An architecture that combines fog and multi-cloud deployments along with Network Coding (NC) techniques, guarantees the needed fault-tolerance for the cloud environment, and also reduces the required amount of redundant data to provide reliable services. In this paper we analyze how NC can actually help to reduce the storage cost and improve the resource efficiency for industrial applications, based on a multi-cloud infrastructure. The cost analysis has been carried out using both real AWS EC2 spot instance prices and, to complement them, prices obtained from a model based on a finite Markov chain, derived from real measurements. We have analyzed the overall system cost, depending on different parameters, showing that configurations that seek to minimize the storage yield a higher cost reduction, due to the strong impact of storage cost.

## 1. Introduction

Industry 4.0, also known as the fourth industrial revolution, fosters the digitalization of industry and manufacturing. This transformation comes mainly through the introduction of Cyber–Physical Systems (CPSs) and the Internet-of-Things (IoT), which give rise to the Industrial IoT (IIoT) and the so-called smart factories [1,2,3]. The usual IIoT scenario is composed of a large number of devices, which send their data to the cloud, where it is processed for real-time or in-rest analysis and visualization. Due to its manifold capabilities, Big Data management among others, cloud computing has become a key enabler of Industry 4.0 [4,5]. However, several IIoT applications, such as system control or robotic guidance, are very sensitive to delay and they usually require immediate decision making. Thus, low latency communications are indispensable for real-time analysis and monitoring, and cloud computing cannot always meet these requirements. For this reason, fog computing [6,7] which is actually considered as an extension of traditional cloud services, is emerging as a promising solution able to provide flexibility and reduce latency [8]. The combination of both fog and cloud-based computing paradigms allows a dynamic workload allocation, to always offer the most suitable service, depending on the needs of each application [9]. In addition to low latency requirements, reliable communications are mandatory for IIoT applications.

Regarding cloud computing, it removes the need of infrastructure investments, yielding a more cost-efficient solution. Nevertheless, its deployment still requires certain expenditure [10], so it becomes important not only ensuring a robust and efficient cloud environment, but making it as cheap as possible. Therefore, the introduction of a new service offered by some cloud providers, referred to as spot instances for Amazon Web Services (AWS) [11], preemtible VM instances for Google Cloud [12], or low-priority VMs for Microsoft Azure [13], may be interesting. These instances offer spare computing capacity at very low prices, with the drawback of not guaranteeing the continuity of the computation and storage resources. Relying the architecture on multiple clouds [14], rather than on a single one, helps to avoid potential service interruptions, making the cloud environment fault-tolerant. Hence, a multi-cloud deployment enhances both data reliability and availability. However, to ensure an acceptable level of reliability, we need to introduce a relevant amount of redundant data, which actually implies a cost increase. The simplest form of redundancy is replication, but using erasure codes we can obtain better storage efficiency. Network Coding (NC) is a type of erasure code with great potential [15,16,17]. The use of NC has been focused on improving communication performance over wireless networks, and its potential to optimize bandwidth and to maximize throughput are well known. NC enables a more robust and stable communication [18], and it also increases throughput and reduces latency [19]. Furthermore, it has been recently proven to be a good solution for distributed storage systems [20], being able to reduce the amount of required redundant data, and thereby diminishing deployment costs.

**Figure 1:**
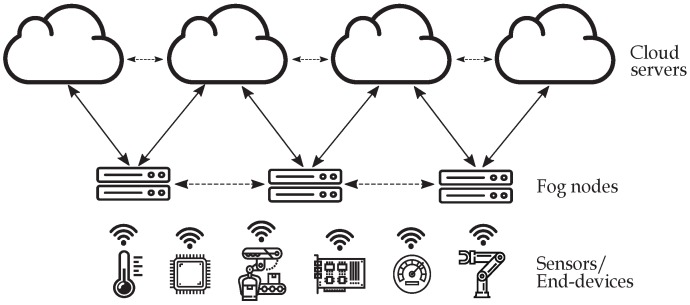
General view of the system, composed of several sensors or IoT devices, one or more fog nodes and multiple cloud servers.

This paper is based on the architecture depicted in Figure 1. The multi-cloud deployment stores data coming from the fog layer, which comprises one or more fog nodes, and takes advantage of the cost reduction of spot instances. The availability depends on the maximum *bid* the user is willing to pay. The system must be highly-reliable, tolerant to service interruptions, while ensuring a continuous data availability. NC, in contrast to Maximum Distance Separable (MDS) codes, as Reed Solomon [21], allows to move over an optimal trade-off curve, which gathers the relationship between the amount of information that each cloud needs to store and the data required to transmit over the system in case a cloud fails [22].

**Figure 2:**
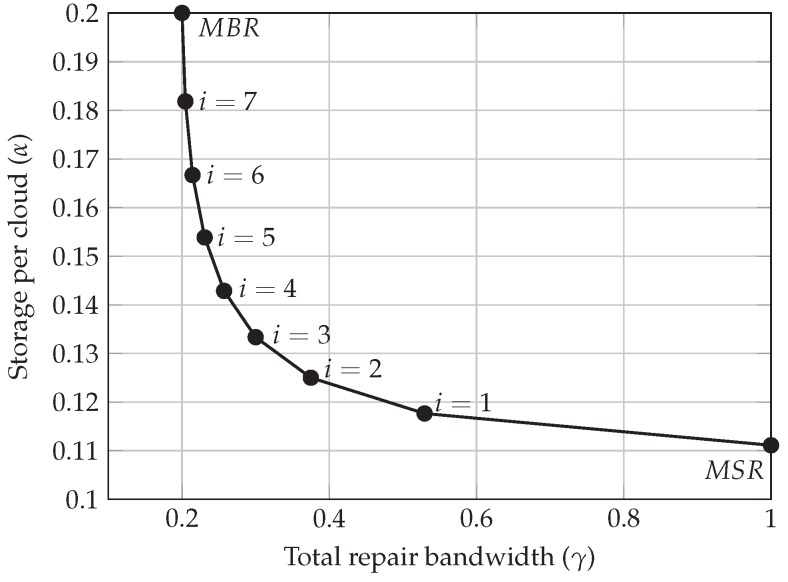
Optimal trade-off curve between storage (α) and repair bandwidth (γ), for k = 9 and M = 1.

We analyze the benefits of using NC-based erasure codes at different points in the trade-off curve (Figure 2), to reduce the cost of developing a reliable distributed storage system, i.e., multi-cloud environment, based on spot instances. The cost analysis has been performed using real AWS spot instance prices as well as a pricing model based on a finite Markov chain [23]. The proposed pricing model enables the generalization of the problem and it also allows to extend it to other price evolutions. Moreover, different system parameters have been modified, such as the bid price, the number of available clouds and interruptions, the data transfer price, or the points of the aforementioned optimal trade-off curve, to study their impact on the proposed scheme.

The remainder of this paper is organized as follows. In Section 2, we review the related work on cloud computing applied to Industry 4.0, and we also introduce some applications in which spot instances and NC are used. We briefly explain the basis of NC with regard to distributed storage systems and the general characteristics of AWS EC2 spot instances. In Section 3 we describe the proposed scenario and we depict the information repair process. We also explain the pricing model that was used during the experiments. In Section 4, we analyze the impact of moving along the trade-off curve on the deployment system cost, as well as the impact of modifying different system parameters. Finally, in Section 5, we conclude the paper, and we outline our future work.

## 2. Related Work

Cloud computing is becoming a cornerstone when it comes to Industry 4.0 or IIoT solutions, and its benefits have been extensively discussed in the literature [24,25,26]. This can also be evinced by the increasing number of ongoing [27,28] and completed [29,30,31] leading European research projects. Authors in [32] develop a cloud-based system to integrate ubiquitous manufacturing applications, and they assess its feasibility for manufacturing chains, human-robot collaboration, and robot’s energy consumption minimization. The authors of [33] propose an IoT-based real-time production logistics synchronization system under cloud manufacturing environment.

Cloud providers like AWS, Microsoft Azure, or Google Cloud offer a wide variety of services, such as storage, computing, databases, Machine Learning, networking, IoT, and security. As previously mentioned, these providers have recently started to offer spare cloud computing capacity at lower prices. Particularly in Amazon Elastic Compute Cloud (Amazon EC2), the available virtual servers or instances, known as *spot instances*, have identical characteristics as regular instances, but with two remarkable differences. First, the hourly price of these instances varies over time based on demand, while the price for the rest of the instances remains static over long periods of time. Second, spot instances do not ensure service continuity, i.e., they are prone to interruptions. Below we enumerate possible reasons for spot instance interruptions [34]:

- *Price:* The user might specify a maximum price per instance/hour, *bid*. If the cost of the resource were greater than this price, the service would be interrupted.
- *Capacity:* A spot instance might suffer an interruption if there were not enough free EC2 capacity to meet the demand for the current instance.
- *Constraints:* When there is an additional constraint, such as a launch group or an availability zone group, the spot instance is terminated when it cannot be satisfied.

With the purpose of simplifying the analysis, only the price-based interruptions are considered in this paper. Spot instances also allow using a warning notification two minutes before the interruption. However, in order to generalize the analysis to different cloud providers that may not offer this service, we would assume that no warning is given prior to an interruption.

Several publications have analyzed the potential of these instances, specially focusing on the cost reduction. For example, authors in [35] conduct a detailed analysis regarding the effect of the location over the overall cost of deploying a spot instance. With the same purpose, some works also characterize and model the spot instance prices. In [36], the authors apply different statistical measures to detect typical pricing patterns, which might help users taking more informed bidding decisions. Authors in [37] use a Neural Network based algorithm with which the future values of spot instances can be predicted, minimizing the overall cost. In [38], both the price and the time between price changes are modeled with a mixed Gaussian distribution. However, pricing patterns change with time, and they might strongly depend on the particular provider; for instance, the authors of [39] state that Amazon’s EC2 prices are typically generated at random, from a dynamic hidden reserve price, rather than being market-driven. Therefore, having an exact model would actually require to statistically re-evaluate the pricing patterns. Opposed to [38], our purpose is not to precisely mimic a particular AWS spot pricing, but rather to build a sensible model reflecting price variations, to allow us carrying out systematic and extensive experiments of the performance of the proposed scheme, so as to yield more accurate and tight results, and to extract more meaningful conclusions.

Many other studies have developed solutions to foster fault tolerance. For instance, the authors of [40,41] propose checkpointing strategies to cope with interruptions of running spot instances. In [42], the authors highlight the need to work on price modeling and to enhance availability. In order to provide a fault-tolerant service, the works discussed in [43,44,45] promoted a distributed system, i.e., a multi-cloud environment.

Random Linear Network Coding (RLNC), the most widespread NC solution, is a particularly valuable tool for this type of environments. It exploits the characteristics of the wireless medium [46,47,48]. It also leverages the properties of distributed storage systems, as demonstrated in [49,50] as well as in the extension of the latter work [51]. In this sense, RLNC enables any node within a network not only to store and forward received packets, but also to recode them into coded packets [15,52]. This is done by linearly combining the packets using randomly chosen coefficients from a finite field $F_{q}$ of size $2^{q}$. The encoding operation of *M* packets can be described as follows:

(1) $$p_{i}^{\prime }=\sum_{j=1}^{M}c_{i,j}\cdot p_{j}$$

where $\left[p_{1},p_{2},\ldots ,p_{M}\right]$ are the original packets, each $p_{i}^{\prime }$ is the resulting coded packet, and $c_{j,i}\in F_{q}$ are the coding coefficients, where for each $p_{i}^{\prime }$, the corresponding set of coding coefficients $\left[c_{i,1},c_{i,2},\ldots ,c_{i,M}\right]$ forms the coding vector. One of the most relevant properties of RLNC is that it is rateless, i.e., we do not need to keep track of the coded packets that have been sent, and the receiver only needs to get any M+ϵ coded packets to recover the original information, where ϵ is a constant that represents the possible linear dependencies received and depends on the finite field used (ϵ≈0 when q≫1).

Regarding reliable and fault-tolerant distributed storage systems, Dimakis et al. showed in [22] the existence of a trade-off curve, illustrated in Figure 2. The curve shows the relationship between the amount of information that is stored in each cloud and the amount of information that needs to be transmitted among the clouds, i.e., between the storage per cloud (α) and the repair bandwidth of the whole system (γ). Theorem 1 presented in [22] gives the feasible points of the optimal trade-off curve. Assuming that a piece of information of size *M* is equally stored over *n* clouds, and that we can recover the information from any k=n−1 clouds (we will focus on k=n−1, but Theorem 1 is valid for any k>1), the following equations represent the (α,γ) points of the trade-off curve:

(2) $$\begin{array}{cc} \alpha & =\frac{M-\frac{(i+1)i}{2k}\gamma }{k-i} \end{array}$$

(3) $$\begin{array}{cc} \gamma & =\frac{2Mk}{(2k-i-1)i+2k} \end{array}$$

where 0≤i≤k−1. The two extreme points of such curve (Figure 2) correspond to the Minimum Storage Regenerating (MSR), where i=0, and Minimum Bandwidth Regenerating (MBR) codes, where i=k−1, whose storage and repair bandwidth are given by (4) and (5), respectively. When MSR codes are used, every cloud stores the same information, each one storing M/k packets. This is the minimum storage needed in order to maintain the fault-tolerance of the system in case another failure occurs, i.e., they behave as optimal MDS codes. However, this implies the transfer of the entire file (*M*). On the other hand, MBR codes allow using less repair bandwidth, which necessarily means that the number of packets to be stored in each cloud is greater.

(4) $$\begin{array}{cc} \left(\alpha_{MSR},\gamma_{MSR}\right) & =\left(\frac{M}{k},M\right) \end{array}$$

(5) $$\begin{array}{cc} \left(\alpha_{MBR},\gamma_{MBR}\right) & =\left(\frac{2M}{k+1},\frac{2M}{k+1}\right) \end{array}$$

Dimakis et al. in [22] perform an extensive theoretical analysis of the trade-off between storage and repair bandwidth for fault-tolerant distributed storage systems. However, it is not applied to any real nor simulated scenario. Based on the previous mathematical characterization, authors in [21,53] study the storage problem, particularly when data should be repaired with the remaining nodes in a fog deployment without newcomer nodes. They also design a practical implementation using a NC implementation and Raspberry Pi devices. Nevertheless, these works only take into account node disconnections, and not the eventual reconnections, and they only analyze MSR and MBR configurations.

Our paper considers cloud interruptions, when the storage price exceeds the bid, as well as cloud appearances, when the price goes again below such bid. The use of NC enables, besides MSR and MBR strategies, to move along all points within the optimal trade-off curve. Hence, we analyze not only these two extreme points, but also other configurations that may be interesting for cost optimization in data storage over multi-cloud scenarios. Moreover, previous research, such as [54], shows the potential of NC to ensure fault tolerance in case of server failures in multi-cloud deployments. To the best of our knowledge, this is the first work proposing NC techniques in order to ensure the reliability of a distributed system prone to failures caused by spot instance interruptions, leading to a novel scenario which benefits from NC features in distributed storage services.

## 3. System Overview

The main objective of this paper is to analyze how NC could foster an efficient distributed storage system, allowing cost reduction, while providing a highly reliable multi-cloud infrastructure, targeting industrial applications. We consider the overall system storage cost as the sum of two components: the first one caused by storing the data, and the second one generated by information transfers. In order to perform this analysis, the storage prices have been obtained from a pricing model that mimics the behavior of real AWS spot instance prices. Our goal, as was mentioned earlier, is to move along the trade-off curve that was discussed in Section 2, by modifying different system parameters, such as the number of clouds and interruptions, or the data transfer cost.

### 3.1. Proposed Scheme

Let one or more fog nodes encode a file of *M* packets, and evenly distribute them into a multi-cloud deployment (see Figure 1). We consider as well that these nodes are able to access the uploaded data at anytime, i.e., the system must ensure continuous data availability. The multi-cloud environment comprises *n* clouds, which depending on the applied RLNC code, store and transfer a given amount of data, both when a cloud is interrupted or when it becomes available again. It is worth highlighting that we only focus on the multi-cloud deployment storage. File distribution and retrieval (from the fog layer) processes are left for our future work.

**Figure 3:**
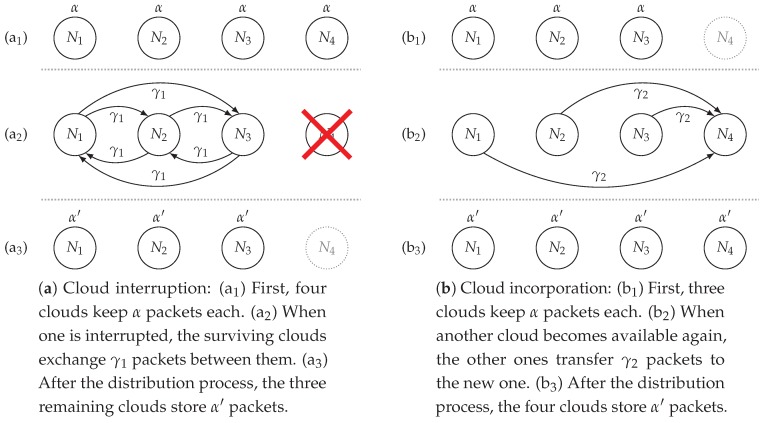
Packet distribution process in case of a cloud (**a**) interruption or (**b**) incorporation.

The availability of one cloud depends on the maximum price (bid) set by the user. When the storage cost of any of the available clouds at a given time exceeds the bid, that cloud is interrupted. On the other hand, when the cost of any cloud goes below the bid, it becomes available again. Therefore, there exist two different situations as shown in Figure 3: (a) cloud interruption, and (b) cloud incorporation, and we jointly refer to them as *transitions*. For the first case, a cloud is lost, so the remaining ones should distribute a given amount of coded packets between them to ensure the system is still reliable to another failure, i.e., moving to the point $\left(\alpha^{\prime },\gamma^{\prime }\right)$; where $\alpha^{\prime }$ represents the number of packets each cloud stores after all of them have been distributed, and $\gamma^{\prime }$ corresponds to those packets that must be transmitted in case another cloud becomes unavailable. This point can be achieved by decreasing the number of surviving clouds by one, k−1, in (2) and (3). When a cloud is interrupted, each of the surviving clouds transfers $\gamma_{1}=\frac{\alpha^{\prime }-\alpha }{k-1}$ recoded packets to the remaining ones, so the total number of packets that need to be transferred over the whole system is $\gamma =\gamma_{1}k\left(k-1\right)$. For the second case, when a cloud becomes available, we would need to transfer $\gamma_{2}=\frac{\alpha^{\prime }}{k}$ packets from each of the other clouds to the incoming one, yielding a total of $\gamma =\alpha^{\prime }$.

### 3.2. Pricing Model

As will be described in Section 4, we have carried out some experiments based on real spot prices. However, the availability of real traces is limited, since the provider only keeps data for three-month windows. Since the provider usually stores one single fee per day, most of our traces have 90 samples. Hence, in order to obtain more insightful results, we will also use a pricing model, which allows us to carry out more repetitive experiments and to better validate the proposed scheme. Javadi et al. used in [38] a mixed Gaussian distribution, whose statistical analysis should be repeated each time to resemble a particular price evolution. Opposed to them, we propose using a Discrete Markov Chain [23], which is a sensible way to model the price evolution within a certain range, leveraging a useful tool to assess the NC benefits on distributed storage systems.

A detailed description of the proposed model is given in Appendix A. In a nutshell, we consider four parameters: (1) the range of fees (spot prices) we would like to consider, $\left[\xi_{\min},\;\xi_{\max}\right]$; (2) the number of states of the model, *N*; (3) the maximum price change in a single transition, $\xi_{\Delta }$; and (4) the slot duration, when the system would update its current price offer. The price corresponding to a particular state *i* is therefore given by: $\xi_{i}=\xi_{\min}+\Delta \cdot i$, where $\Delta =\frac{\xi_{\max}-\xi_{\min}}{N-1}$. In addition, the transition probabilities are established to include some ‘memory’ within the model, so that moving to states closer to the current one is more likely.

Based on how fast it would mimic price variations (as it is discussed in Appendix A), we have used two particular configurations of the proposed model. In the first one, we restrict state transitions so that they can only go to states that are next to the current one, i.e., $\xi_{\Delta }=\Delta$. We refer to this first configuration as *Slow Price Model* (SPM). Being δ≥0 the ‘gap’ between states *i* and *j*: $\delta =\left|i-j\right|$, the transition probability between states, $p_{ij}$, can be defined as follows:

(6) $$p_{ij}=\left\{\begin{array}{cc} 0 & \delta >1\\ \frac{1}{2H\left(2\right)-1}\cdot \frac{1}{\delta +1}\; & i=1\ldots N-2,\;\;\delta \leq 1\\ \frac{1}{H\left(2\right)}\cdot \frac{1}{\delta +1} & i=0,N-1,\;\;\delta \leq 1 \end{array}\right.$$

where H(n) is the *n*th harmonic number. The proof is given in Appendix A.

In the second one, we do not impose any restriction on the price change at a state transition event, so $\xi_{\Delta }\geq \xi_{\max}-\xi_{\min}$, and we thus, consider any potential transition. We will refer to this scheme as *Fast Price Model* (FPM), being the following its transition probability:

(7) $$p_{ij}=\frac{1}{H(i+1)+H(N-i-1)}\cdot \frac{1}{\delta +1}$$

The proof of $p_{ij}$ of the model and its configurations is given in Appendix A.

**Figure 4:**
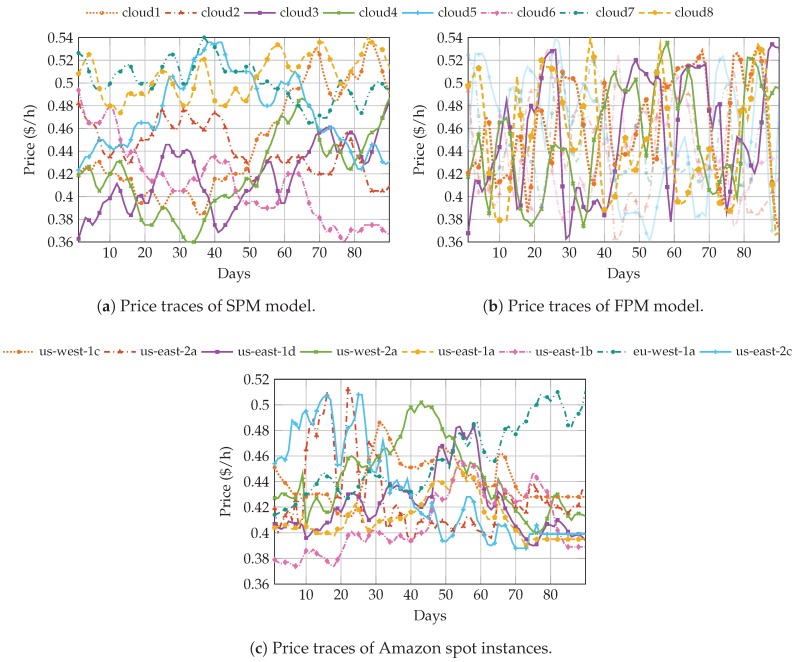
Pricing of 8 clouds for the (**a**) SPM pricing model, (**b**) FPM pricing model, and (**c**) real spot instance pricing history.

In order to assess the validity of the proposed approach, Figure 4a,b show the particular evolution of the spot prices offered by 8 clouds, where the range of fees were selected to resemble the real values we obtained from Amazon: [0.36,0.54]$/h. We configured the chain with N=12 states, and we simulated 90 daily slots.

AWS EC2 provides a variety of instances, each of which offers different computing, memory, and storage capabilities. The offered instances can be of general purpose, computing optimized, memory optimized, storage optimized, or for accelerated computing [55]. The type that was selected in this work is *i3*, a Non-Volatile Memory Express (NVMe) SSD-based storage instance, optimized for low latency applications, particularly suitable for fog computing based industrial applications. It offers very high random I/O performance, and high sequential read throughput [56].

Spot instance prices of 90 days can be obtained by accessing the console of AWS EC2 [57], from which we have acquired the pricing history, specifically from 2018-03-13 to 2018-06-10. Since not all instances have the same number of samples, we processed them calculating the average hourly price for each of the 90 days. Figure 4c shows the evolution of such prices, in ($/h), for i3.4xlarge Linux/UNIX OS instances corresponding to different regions. In order to estimate the storage cost, which depends on the number of stored packets, we have obtained the hourly cost per GB($/GBh), by checking the storage capacity of i3.4xlarge instances [56].

Figure 4a,b show the result of SPM and FPM, respectively. As can be noted, the evolution of the fees mimics quite well the behavior of the real traces, since for similar periods of time, the whole range of price values was covered. We can clearly see the differences between the two configurations, in particular considering the price change pace. By comparing with the real traces, we can conclude that these two ‘extreme’ cases are able to capture the whole range of behaviors that were observed in real systems. Traces obtained with SPM are slightly less variable than the real ones. However, there are some instances, for instance *cloud1* in Figure 4a, that cover a reasonably wide range of prices. On the other hand, FPM shows a price evolution pace that is faster than the one observed for the real ones, with rather abrupt price changes. However, we could also observe that in some cases, for instance *us-east-2c*, these fast price changes were also observed in the Amazon price traces (we highlight four price traces to facilitate their visualization).

## 4. Results and Discussion

This paper focuses on analyzing the cost of deploying a reliable multi-cloud environment targeted to data storage. Several simulations have been carried out in order to understand the impact of modifying different parameters involving the distributed storage system. In order to obtain more insightful results, statistically tight, we have conducted a large number of independent experiments over different scenarios, generated exploiting the pricing model that was described in Section 3.2. In addition, we also compare the results with those obtained with real AWS spot instances. We generate traces entailing 90-day runs of the multi-cloud deployment, and we have simulated 200 scenarios of 10 clouds with each model.

We have carried out the experiments with a file of 512 KB. The overall cost of storing a file in the distributed system corresponds the sum of the actual storage cost ($/GBh) and the transfer cost ($/GB). The former depends on the particular region, as well as on the date when the instance is executed. The average storage price for i3.4xlarge instances [58] is approximately 0.03 $/GBh. We have applied multiple transfer prices: 0.01 $/GB (usual prices in AWS EC2 for the United States and Europe vary within the 0.01–0.02 $/GB range), 0.05 $/GB, and 0.1 $/GB (similar to Asian transfer prices), and 0.5 $/GB. The latter is considerably greater than the prices offered by Amazon, since other cloud providers might use rather different strategies. Moreover, the future price evolution is very uncertain, although the storage price is believed to reduce, while being more volatile, with changes occurring more frequently (i.e., on a hourly, rather than daily, basis).

We are interested in studying the behavior of different NC codes. Hence, we have applied four points of the optimal trade-off curve. First, we analyze the two extreme configurations, MSR and MBR. In addition, the use of the two closest points to MSR (i=1 and i=2) could be particularly beneficial, since by slightly increasing the amount of stored packets at each cloud, the repair bandwidth is strongly reduced, recall Figure 2.

### 4.1. Simulations Using the Pricing Model

First, we have analyzed the variation of the overall cost, by increasing the maximum acceptable price (bid), which is specified by the user, using the previous setup, focusing on the aforementioned four points: MSR, MBR, i=1, and i=2. Besides the overall cost, we also represent the average number of available clouds during the simulation for each bid, i.e., those whose prices stay below the bid.

**Figure 5:**
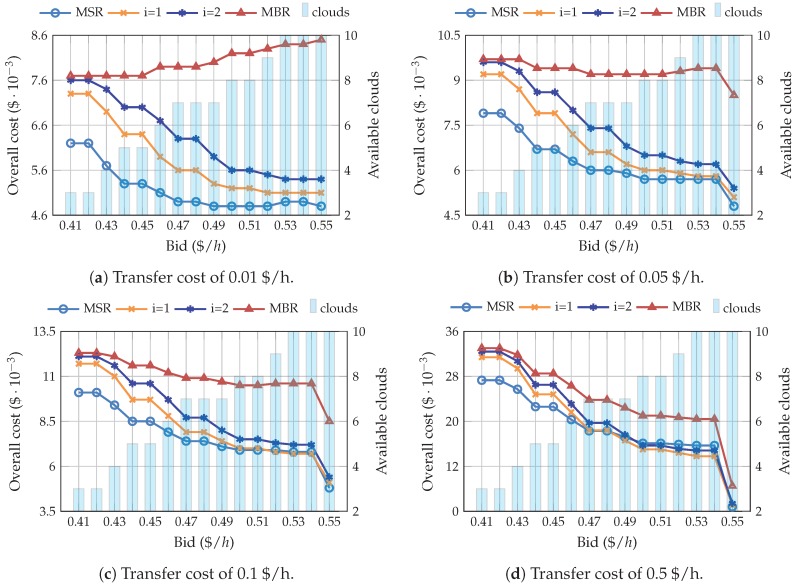
Relationship between the overall cost, the bid, and the number of available clouds, for pricing scenarios generated with the SPM configuration.

Figure 5 clearly shows that, even with high transfer prices, MBR is the most expensive code to use. Nonetheless, as could have been expected, if the transfer price is increased, the difference with the other configurations gets lower. This is because, as explained in Section 2, MBR requires less repair bandwidth and the benefits of this code are thus, enhanced when the transfer cost increases. However, it is generally not more economical than MSR. It can also be noted that setting higher bids decreases the overall cost, yielding a greater number of available clouds. Therefore, the more clouds available, the more cost-efficient the system becomes. Furthermore, the overall cost shows a drastic decrease with a bid of 0.55 $/h due to the lack of transitions, i.e., interruptions and incorporations, avoiding costs due to data transfer.

Note that the file is stored during 90 days, while data is only transferred when a transition occurs. Hence, although the transfer price (≈0.01 $/GB) is one order of magnitude higher than the storage price (≈0.003 $/GBh), the latter has a greater impact on the overall cost. For this reason, MSR shows the best behavior. However, it is important to highlight that configurations i=1 and i=2 could be more profitable than MSR at some cases, for instance i=1 with 0.49 $/h bid in Figure 5c. As transfer prices increase, i=2 also outperforms the cost-efficiency of MSR, recall Figure 5d. This happens because, as shown in Figure 2, these points allow to considerably reduce the transfer cost, by slightly increasing the stored amount of information. Therefore, the benefits are even more noticeable as the transfer price increases. Considering the above, it can be seen in Figure 5a that, opposed to the rest of the codes, MBR shows an increasing trend. This happens because when using MBR, the system needs to store the whole file, regardless of the bid, and the price of the storage increases with the bid.

**Figure 6:**
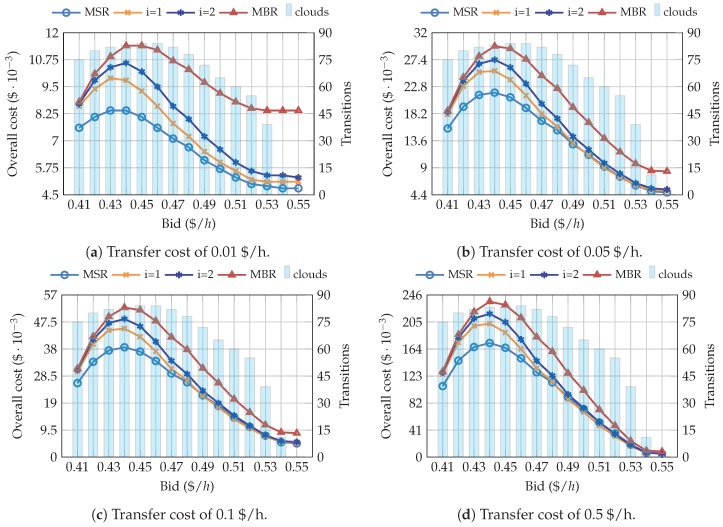
Relationship between the overall cost, the bid, and the number of transitions, for pricing scenarios generated with the FPM configuration.

FPM shows a similar behavior to SPM regarding the average number of available clouds, which increase with the bid. Hence, in order to better understand such behaviors, Figure 6 does not only show the overall cost, but it also includes the number of transitions. The results seen for the FPM are not like those obtained with SPM. In this case, the overall cost has a decreasing trend, but only above a certain bid, which corresponds to the upper half of the model’s price range [0.45, 0.54] $/h. However, it can be clearly observed that the cost drastically increases for a bid of approximately 0.45 $/h, due to the great number of transitions. Regarding the NC codes, as was seen for the SPM, MBR is again the most expensive scheme, with the difference being lower as the transfer price increases. Points i=1 and i=2 are slightly cheaper than MSR for bids higher than 0.49 $/h and a transfer price of 0.05 $/h and 0.1 $/h, respectively. Moreover, it is worth stressing that the storage cost is not constant, since it depends on the number of currently available clouds, and the optimal point of the trade-off curve can be different for each scenario, and might even change over time.

**Figure 7:**
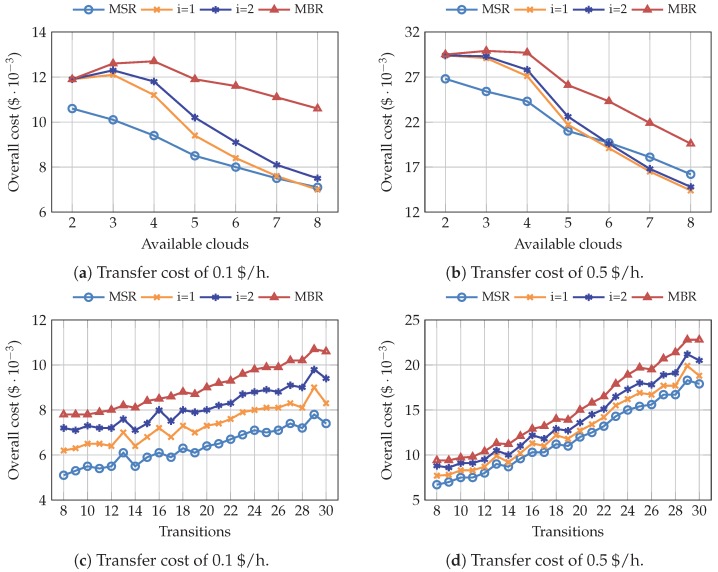
Overall cost of pricing scenarios generated with SPM, depending on the number of average available clouds and the number of transitions.

We have carried out multiple simulations to analyze the impact of the average number of available clouds and the number of transitions on the overall cost. The results are depicted in Figure 7. They correspond to a configuration where we established a fixed bid (0.45 $/h), and we have processed the scenarios depending on the average number of available clouds and the number of transitions, respectively. Taking into account that both SPM and FPM show a similar behavior regarding these two parameters, we only discuss the results obtained for the SPM configuration.

Figure 7a,b show the overall cost when different number of clouds are available. As previously seen in Figure 5, the total cost decreases as long as more clouds become available. It can be clearly observed that MBR is the most expensive code, while MSR yields the best results. However, it can be also noted that, for greater transfer prices, the distance between both codes is significantly reduced. The intermediate points, i=1 and i=2, outperform MSR when higher transfer prices are applied. This leads to a considerable increase in the transfer price, which is much higher than those currently offered by AWS.

The relationship between the overall cost and the number of transitions is depicted in Figure 7c,d. In this case, we have filtered the scenarios considering the average number of transitions, i.e., interruptions and cloud additions. As was expected, the overall cost trend is increasing: the more the transitions occur, the greater the overall cost of the distributed system. Both figures show that the use of NC codes corresponding to lower points of the optimal trade-off curve might reduce the overall cost, with MSR and MBR being the most and less economical choices, respectively. Since the transfer cost increases as the number of transitions gets higher, the difference observed when using different codes is less relevant, especially when the number of transitions is low. Finally, Figure 7d shows that there is a significant difference between MBR and the other codes, so the transfer price should be considerably higher for this code to be advantageous.

### 4.2. Simulations Using the Spot Pricing History

In this section, we carry out simulations using a scenario built from the real AWS EC2 spot instance prices, and we compare the results with those seen for the pricing models, which would also validate them. As we have already mentioned, the available set of data acquired from Amazon is limited and we are, thus, not able to create a large number of setups, as we did with the models. We run the simulations over the scenario illustrated in Figure 4c, which consists of 8 clouds or instances, whose prices oscillate between [0.45,0.54]$/h.

**Figure 8:**
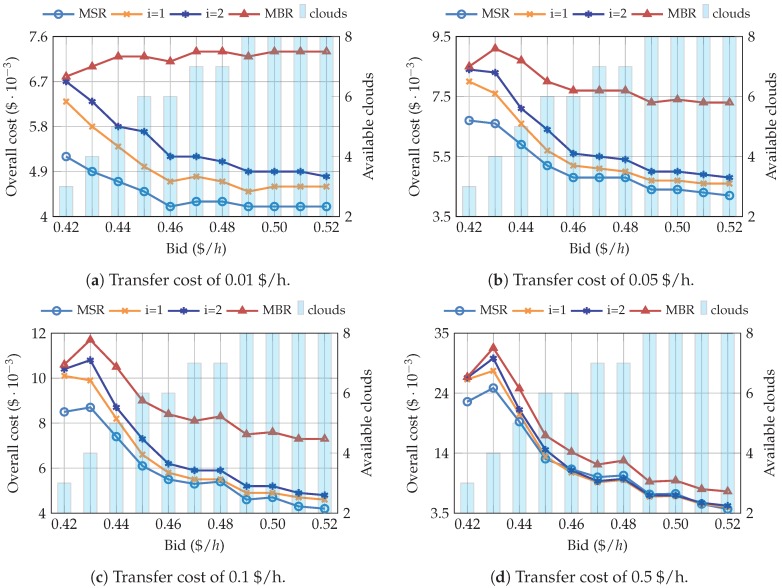
Relationship between the overall cost, the bid, and the number of available clouds, for AWS spot pricing scenarios.

The relationship between the overall cost, the bid price and the average number of available clouds is depicted in Figure 8. The behavior is similar to SPM, and the overall cost tends to decrease, although in this case, we can see that the slope is more steep, since with lower bids almost all clouds are available. Regarding the NC codes, the results are similar to those obtained with the two pricing model configurations. The cost of MBR is considerably higher than the rest of the codes (i=1, i=2, and MSR). Similar to the previous results, when the transfer price increases, the difference is considerably reduced. Furthermore, for this specific case, using AWS EC2 spot prices, i=1 and i=2 only become more cost-efficient than MSR when the transfer price equals 0.5 $/h.

## 5. Conclusions

In this paper, we analyze the potential of NC-based techniques to provide a cost-efficient distributed storage system, relying upon failures. We have particularly focused on architectures for industrial applications, where there exists an interaction between the fog layer and a multi-cloud deployment. The system takes advantage of the cost reduction, both in storage and computing capacity, offered, in this particular case, by Amazon EC2 *spot instances*. The use of this type of services could cause some interruptions, among other factors, when the resource price surpasses the maximum *bid* the user is willing to pay. NC can be beneficial to avoid these interruptions, guaranteeing a highly reliable system, while ensuring continuous data availability. In addition to the interruptions, we have also considered the possibility of a cloud becoming available again.

The fault tolerance of the multi-cloud environment can be achieved by storing the correct amount of data on each of the clouds (α), which also determines the amount of data that needs to be transferred among them (γ) to become tolerant to another eventual failure. Thus, the storage cost is directly linked to these two parameters, and NC allows to move along the optimal trade-off curve that relates them. We have extensively analyzed four points of this curve that can be interesting for the storage cost reduction: the two extreme points (MSR and MBR) as well as two intermediate points (i=1 and i=2).

In order to carry out this analysis, we have acquired real spot instance prices. However, as the available price data set was limited, we have also developed a pricing model based on a finite Markov Chain that reflects realistic price variations, similar to those of AWS. This model has allowed us to carry out a more complete set of simulations, achieving more insightful results. It would also enable to generalize the problem to possible future price evolution, as well as to other potential cloud providers. We have used two particular configurations: (a) SPM, where state transitions are restricted to the adjacent states, and (b) FPM, in which we do not impose any restriction.

We have performed multiple simulations to analyze the impact of the NC codes on the total cost, modifying different system parameters: the bid, the transfer prices, the average number of available clouds, and the number of interruptions.

The main results are the following: (1) MBR stands as the most expensive code, while MSR is generally the cheapest one. However, there might be some configurations, with high transfer prices, yielding lower costs. (2) The overall cost decreases as more clouds become available, and it increases when more transitions are observed during the experiment. (3) The overall cost observed with the proposed SPM mimics quite well the one obtained when using real traces.

In addition, the results show that a sensible approach would be to establish the highest bid, to minimize the overall cost. However, real spot instance prices are highly dynamic and it is therefore difficult to predict their exact behavior.

In our future work, we would like to generalize this setup, including multiple fog nodes and considering the use of NC for communication purposes, and not only for storage. Besides, by collecting more price data from AWS spot instances or similar providers, we could tune the proposed model, training it more appropriately, to leverage more accurate simulations. On the other hand, since low-latency is a stringent requirement of Industrial IoT environments, we will also extend the proposed scheme to ensure low-latency communications in a fog to multi-cloud environment, targeting delay-sensitive applications. For that we will explore exploiting the frameworks provided by projects that are working in the Industry 4.0 realm [59,60].

## Figures and Tables

**Figure A1:**
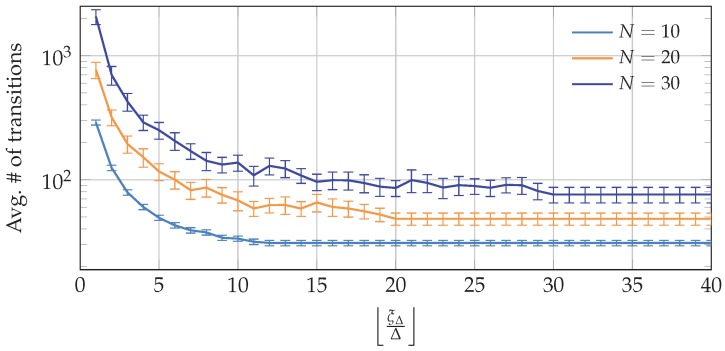
Price model speed, as the number of transitions that are required to cover the whole price range. The graph shows the average value as well as the 95% confidence interval, after 100 independent experiments.

**Figure A2:**
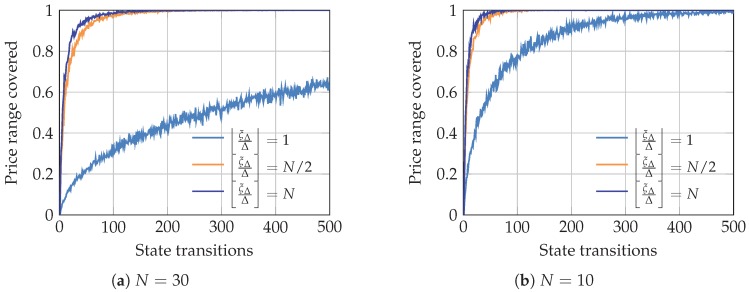
Price model speed, as the covered price range after Vs. the number of state transitions. The graph shows the average value after 100 independent experiments.

## Appendix A. Spot Pricing Model

In order to model the evolution of real spot prices, we propose the use of a Discrete Markov Chain. We take as inputs the maximum and minimum fees we want to consider, $\xi_{\max}$ and $\xi_{\min}$, respectively, as well as the number of states, *N*. Hence, the chain moves within states ∈0…N−1. Each state *i* would yield a certain spot price, which can be obtained as: $\xi_{i}=\xi_{\min}+i\cdot \Delta$, where Δ corresponds to the price hop between consecutive states: $\Delta =\frac{\xi_{\max}-\xi_{\min}}{N-1}$.

The model is generic enough, and can be thus, used for different time scales. We consider that at every slot (which might be a second, minute, hour or even day), there is a state change, which is based on the corresponding transition probabilities, $p_{ij}$, which are defined as the probability that the chain moves from current state *i* to *j*.

In order to mimic a more realistic price evolution, we include some *memory* within the proposed model. For that, we decrease the transition probability when the distance (gap) between the current and the destination states gets higher. If we define $\delta =\left|i-j\right|$ as such difference (δ≥0), then:

(A1) $$p_{ij}=\frac{\kappa }{\delta +1}$$

where κ is a normalizing constant so that the sum of all transition probabilities equals 1.

In addition, we also consider the maximum price variation that we may have in a transition, so that bid changes are within a certain range. For that, we establish the following parameter: $\xi_{\Delta }$, which is defined as the maximum bid change that may happen in a single transition:

(A2) $$p_{ij}=0\;\mathrm{if}\;\left|j-i\right|>\left\lfloor \frac{\xi_{\Delta }}{\Delta }\right\rfloor$$

Hence, for a particular current state *i*, we can distinguish four different cases, in which the value of κ would be different:

(i) $i<\left\lfloor \frac{\xi_{\Delta }}{\Delta }\right\rfloor$ and $N-1-i<\left\lfloor \frac{\xi_{\Delta }}{\Delta }\right\rfloor$: in this case the transitions are not restricted, and any state can be reached from *i*, regardless of its value.
  (A3) $$\begin{array}{cc} \sum_{j=0}^{N-1}p_{ij}=\sum_{j=0}^{i}p_{ij}+\sum_{j=i+1}^{N-1}p_{ij}=\underset{j=i\ldots 0}{\underset{︸}{\sum_{\delta =0}^{i}\frac{\kappa }{\delta +1}}}+\underset{j=i+1\ldots N-1}{\underset{︸}{\sum_{\delta =1}^{N-1-i}\frac{\kappa }{\delta +1}}} & =\kappa \left[\sum_{t=1}^{i+1}\frac{1}{t}+\sum_{t=2}^{N-i}\frac{1}{t}\right]\\ & =\kappa \left[H(i+1)+H(N-i)-1\right] \end{array}$$
(ii) $i<\left\lfloor \frac{\xi_{\Delta }}{\Delta }\right\rfloor$ and $N-1-i\geq \left\lfloor \frac{\xi_{\Delta }}{\Delta }\right\rfloor$: the transitions to states j>i are restricted, since we cannot reach a state *j*, in which $j-i>\left\lfloor \frac{\xi_{\Delta }}{\Delta }\right\rfloor$. Such transition probabilities are 0, according to (A2) and are thus, not considered in the sum.
  (A4) $$\begin{array}{cc} \sum_{j=0}^{N-1}p_{ij}=\sum_{j=0}^{i}p_{ij}+\sum_{j=i+1}^{\left\lfloor \frac{\xi_{\Delta }}{\Delta }\right\rfloor +i}p_{ij}=\underset{j=i\ldots 0}{\underset{︸}{\sum_{\delta =0}^{i}\frac{\kappa }{\delta +1}}}+\underset{j=i+1\ldots \left\lfloor \frac{\xi_{\Delta }}{\Delta }\right\rfloor +i}{\underset{︸}{\sum_{\delta =1}^{\left\lfloor \frac{\xi_{\Delta }}{\Delta }\right\rfloor }\frac{\kappa }{\delta +1}}} & =\kappa \left[\sum_{t=1}^{i+1}\frac{1}{t}+\sum_{t=2}^{\left\lfloor \frac{\xi_{\Delta }}{\Delta }\right\rfloor +1}\frac{1}{t}\right]\\ & =\kappa \left[H\left(i+1\right)+H\left(\left\lfloor \frac{\xi_{\Delta }}{\Delta }\right\rfloor +1\right)-1\right] \end{array}$$
(iii) $i\geq \left\lfloor \frac{\xi_{\Delta }}{\Delta }\right\rfloor$ and $N-1-i<\left\lfloor \frac{\xi_{\Delta }}{\Delta }\right\rfloor$: transitions to states j<i are restricted, since we cannot reach a state *j*, in which $i-j>\left\lfloor \frac{\xi_{\Delta }}{\Delta }\right\rfloor$. Such transition probabilities are 0, according to (A2) and are thus, not considered in the sum.
  (A5) $$\begin{array}{cc} \sum_{j=0}^{N-1}p_{ij}=\sum_{j=\left\lfloor \frac{\xi_{\Delta }}{\Delta }\right\rfloor -i}^{i}p_{ij}+\sum_{j=i+1}^{N-1}p_{ij}=\underset{j=i\ldots \left\lfloor \frac{\xi_{\Delta }}{\Delta }\right\rfloor -i}{\underset{︸}{\sum_{\delta =0}^{\left\lfloor \frac{\xi_{\Delta }}{\Delta }\right\rfloor }\frac{\kappa }{\delta +1}}}+\underset{j=i+1\ldots N-1}{\underset{︸}{\sum_{\delta =1}^{N-1-i}\frac{\kappa }{\delta +1}}} & =\kappa \left[\sum_{t=1}^{\left\lfloor \frac{\xi_{\Delta }}{\Delta }\right\rfloor +1}\frac{1}{t}+\sum_{t=2}^{N-i}\frac{1}{t}\right]\\ & =\kappa \left[H\left(\left\lfloor \frac{\xi_{\Delta }}{\Delta }\right\rfloor +1\right)+H\left(N-i\right)-1\right] \end{array}$$
(iv) $i\geq \left\lfloor \frac{\xi_{\Delta }}{\Delta }\right\rfloor$ and $N-1-i\geq \left\lfloor \frac{\xi_{\Delta }}{\Delta }\right\rfloor$: in this case both transitions, to j<i and j>i, are restricted.
  (A6) $$\begin{array}{cc} \sum_{j=0}^{N-1}p_{ij}=\sum_{j=\left\lfloor \frac{\xi_{\Delta }}{\Delta }\right\rfloor -i}^{i}p_{ij}+\sum_{j=i+1}^{\left\lfloor \frac{\xi_{\Delta }}{\Delta }\right\rfloor +i}p_{ij}=\underset{j=i\ldots \left\lfloor \frac{\xi_{\Delta }}{\Delta }\right\rfloor }{\underset{︸}{\sum_{\delta =0}^{\left\lfloor \frac{\xi_{\Delta }}{\Delta }\right\rfloor }\frac{\kappa }{\delta +1}}}+\underset{j=i+1\ldots \left\lfloor \frac{\xi_{\Delta }}{\Delta }\right\rfloor +i}{\underset{︸}{\sum_{\delta =1}^{\left\lfloor \frac{\xi_{\Delta }}{\Delta }\right\rfloor -i}\frac{\kappa }{\delta +1}}} & =\kappa \left[\sum_{t=1}^{\left\lfloor \frac{\xi_{\Delta }}{\Delta }\right\rfloor +1}\frac{1}{t}+\sum_{t=2}^{\left\lfloor \frac{\xi_{\Delta }}{\Delta }\right\rfloor +1}\frac{1}{t}\right]\\ & =\kappa \left[2H\left(\left\lfloor \frac{\xi_{\Delta }}{\Delta }\right\rfloor +1\right)-1\right] \end{array}$$
  where H(n) is the $n^{\mathrm{th}}$ harmonic number, i.e., the sum of the *n* first terms of the harmonic progression: $H_{n}=\sum_{i=1}^{n}\frac{1}{i}$.

Since the sum of all transition probabilities equals 1 for the four identified cases, (A3)–(A6), we can finally obtain that:

(A7) $$p_{ij}=\left\{\begin{array}{cc} 0 & \delta >\left\lfloor \frac{\xi_{\Delta }}{\Delta }\right\rfloor ,\;\;\delta >1\\ \frac{1}{H(i+1)+H(N-i)-1}\cdot \frac{1}{\delta +1} & i<\left\lfloor \frac{\xi_{\Delta }}{\Delta }\right\rfloor ,\;\;i>N-1-\left\lfloor \frac{\xi_{\Delta }}{\Delta }\right\rfloor ,\;\;\delta \leq 1\\ \frac{1}{H\left(i+1\right)+H\left(\left\lfloor \frac{\xi_{\Delta }}{\Delta }\right\rfloor +1\right)-1}\cdot \frac{1}{\delta +1}\; & i<\left\lfloor \frac{\xi_{\Delta }}{\Delta }\right\rfloor ,\;\;i\leq N-1-\left\lfloor \frac{\xi_{\Delta }}{\Delta }\right\rfloor ,\;\;\delta \leq 1\\ \frac{1}{H\left(\left\lfloor \frac{\xi_{\Delta }}{\Delta }\right\rfloor +1\right)+H\left(N-i\right)-1}\cdot \frac{1}{\delta +1} & i\geq \left\lfloor \frac{\xi_{\Delta }}{\Delta }\right\rfloor ,\;\;i>N-1-\left\lfloor \frac{\xi_{\Delta }}{\Delta }\right\rfloor ,\;\;\delta \leq 1\\ \frac{1}{2H\left(\left\lfloor \frac{\xi_{\Delta }}{\Delta }\right\rfloor +1\right)-1}\cdot \frac{1}{\delta +1} & i\geq \left\lfloor \frac{\xi_{\Delta }}{\Delta }\right\rfloor ,\;\;i\leq N-1-\left\lfloor \frac{\xi_{\Delta }}{\Delta }\right\rfloor ,\;\;\delta \leq 1 \end{array}\right.$$

As can be inferred, by modifying two parameters of the proposed pricing model, namely the maximum price variation and the number of states, we can tweak its configuration, leading to different behaviors. In order to better understand the impact of these two parameters over the price evolution, we have carried out an extensive simulation campaign. We first define the model ‘speed’ as the number of transitions that are required to cover the whole price range, i.e., to visit states 0 and N−1, when starting from a random initial state *i*. Such initial state is randomly selected according to the stationary distribution of the corresponding Markov chain, which is ergodic, since any state can be reached from any other one. Hence, there exists a unique stationary state probability distribution, defined by the column vector $\bar{\pi }=\left[\pi_{0},\pi_{1}\ldots \pi_{N-1}\right]$, which satisfies:

(A8) $$\begin{array}{c} \bar{\pi }=P\cdot \bar{\pi } \end{array}$$

(A9) $$\begin{array}{c} \sum_{i=0}^{N-1}\pi_{i}=1 \end{array}$$

where P is the transition matrix, defined by the transition probabilities $p_{ij}$. $\bar{\pi }$ can be thus, obtained by solving (A9), which has a unique solution, according to its aforementioned ergodicity.

In Figure A1 we can see such ‘speed’, as we increase the maximum price variation of a single transition, $\xi_{\Delta }$, and for different number of states, *N*. As can be seen, when $\frac{\xi_{\Delta }}{\Delta }=1$ (i.e., the chain can be just go to states that are next to the current one), the number of transitions that are required to cover the whole price range is rather high. Afterwards, it starts to decrease (the model becomes faster), until it stabilizes, when the configuration does not impose any constraint on the number of states that can be jumped on a single transition, i.e., $\frac{\xi_{\Delta }}{\Delta }\geq N$.

In addition, Figure A2 shows an alternate way of assessing the model speed. We fix the value of $\frac{\xi_{\Delta }}{\Delta }$, and we plot the evolution of the price range that has been covered after a certain number of transitions. As can be seen, the figure yields a similar conclusion as the previous one. When we restrict transitions to happen between consecutive states, $\frac{\xi_{\Delta }}{\Delta }=1$, the number of changes that are required to cover a wide price range is quite high, especially when the number of states is greater. When we increase $\frac{\xi_{\Delta }}{\Delta }$, there is a remarkable reduction on the number of transitions, but the figure evinces that such reduction is much less noticeable for further increases, i.e., from $\frac{N}{2}$ to *N*.

Thanks to the previous analysis, we could establish sensible configurations for the proposed model. For instance, if we would like to mimic a slow price evolution, we should use a low value of $\frac{\xi_{\Delta }}{\Delta }$, but this would also imply having a small number of states, since otherwise, the whole price range would not be likely covered.
